# Hybrid Tracheal Stent Ingestion in Malignant Central Airway Stenosis: A Case Report

**DOI:** 10.1002/rcr2.70329

**Published:** 2025-09-02

**Authors:** Yuya Ishikawa, Hironori Ishibashi, Yukitaka Sato, Yuri Sumi, Ayaka Asakawa, Ryo Wakejima, Kenichi Okubo

**Affiliations:** ^1^ Department of Thoracic Surgery Institute of Science Tokyo Tokyo Japan

**Keywords:** hybrid stent, malignant central airway stenosis, stent ingestion, stent migration, tracheal stent

## Abstract

Airway stents provide symptomatic relief in malignant central airway stenosis, but carry the risk of migration, which may result in potentially lethal stent ingestion. A 22‐year‐old man underwent hybrid stent placement for tracheobronchial stenosis caused by an undiagnosed large mediastinal mass. Within 3 weeks after placement, following initial chemotherapy for provisionally diagnosed B‐cell lymphoma, the stent migrated and was ingested. The stent was successfully retrieved endoscopically. This is the first reported case of hybrid tracheal stent ingestion. Early surveillance is warranted in patients with anticipated tumour regression.

## Graphical abstract

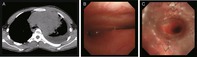

## 1 Introduction

Airway stents are commonly used to treat malignant central airway stenosis and to improve symptoms and respiratory function. However, complications such as infection, granulation tissue formation, and migration remain a concern. Silicone stents are more prone to migration than self‐expandable metallic stents. Hybrid stents have been developed to reduce this risk; however, migration can occur, particularly in anatomically unstable situations. This report describes a rare case of hybrid stent ingestion and emphasises the importance of surveillance in patients with expected rapid anatomical changes.

## 2 Case Report

**FIGURE 1:**
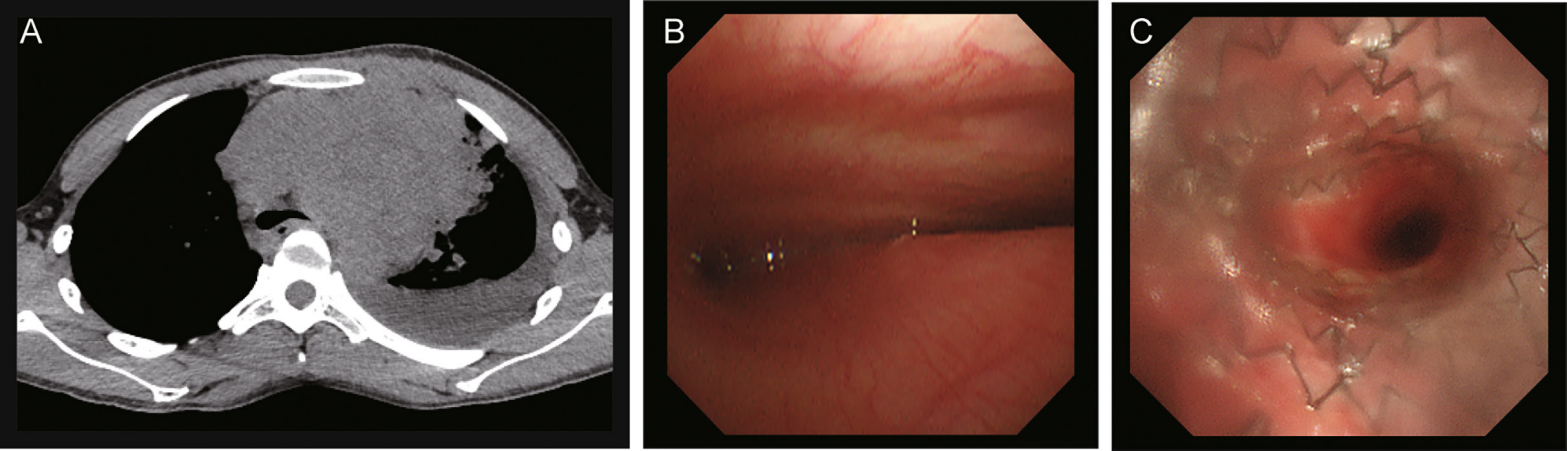
Findings before and after stent placement. (A) Non‐contrast chest computed tomography (CT) at presentation showing an anterior mediastinal mass causing external compression and complete obstruction of the left main bronchus. (B) Bronchoscopic view at the carina level revealing significant luminal narrowing from extrinsic compression, with both main bronchi obscured. (C) Bronchoscopic image demonstrating successful hybrid stent deployment with restored patency of the right main bronchus.

A 22‐year‐old man presented with dyspnea and cough that worsened in the supine position, along with a palpable subcutaneous mass on the anterior chest. Computed tomography (CT) of the chest revealed a 137‐mm anterior mediastinal mass with tracheal stenosis and left main bronchial stenosis due to extrinsic compression (Figure 1A,B). Tracheal stenosis was observed 50 mm proximal to the carina, narrowing from a normal diameter of 15–4 mm at the most stenotic point. A needle biopsy of the subcutaneous mass was performed on the day of referral. Given the risk of airway stenosis before a definitive diagnosis and treatment, a tracheal stent was placed on hospital day 5 to secure the airway and maintain the patency of the right main bronchus. The AERO stent (Merit Medical Systems, South Jordan, UT, USA), a fully covered self‐expanding metallic stent, was chosen because of its strong expansile force and removability. Based on the stenotic length and airway diameter, a 60‐mm‐long, 16‐mm‐diameter stent was selected (Figure 1C).

A presumptive diagnosis of B‐cell lymphoma was made, and R‐CHOP (rituximab, cyclophosphamide, doxorubicin, vincristine, and prednisone) chemotherapy was initiated on postprocedural Day 5. The patient experienced improved oxygenation and resolution of his positional symptoms. He was discharged without oxygen support on postprocedural Day 21.

**FIGURE 2:**
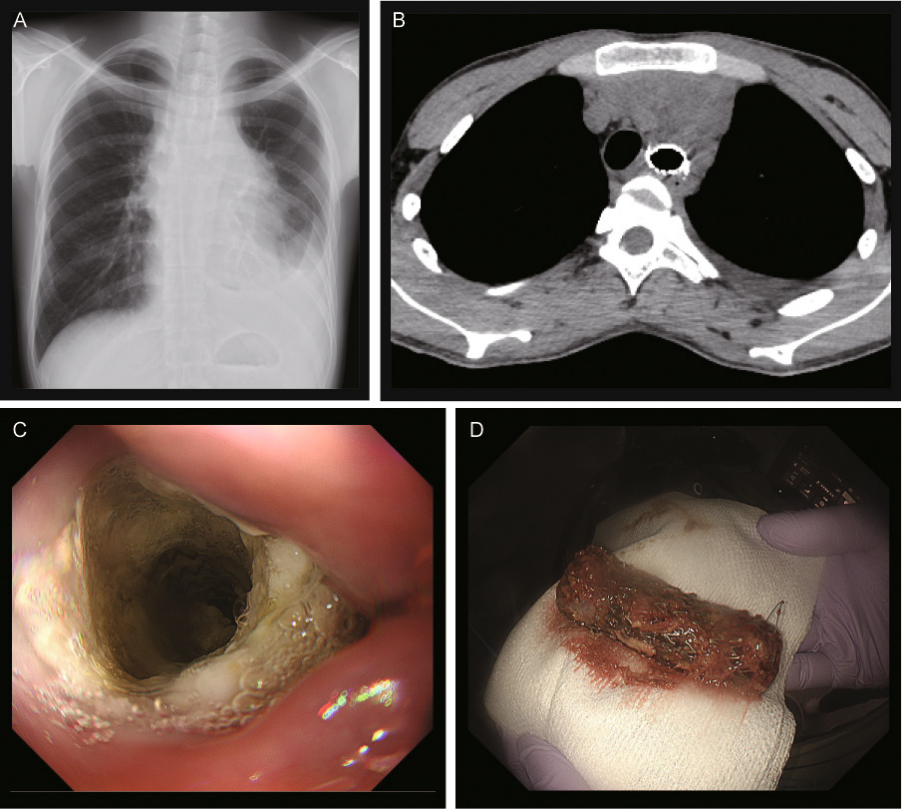
Stent ingestion findings. (A) Chest x‐ray on readmission showing the migrated stent located adjacent to the trachea. (B) Non‐contrast chest CT showing the tracheal stent within the oesophagus. (C) Upper gastrointestinal endoscopy revealing the hybrid stent lodged in the oesophagus. (D) The retrieved hybrid stent, which remained intact and undamaged after endoscopic removal.

After a final diagnosis of primary mediastinal large B‐cell lymphoma, the patient was readmitted 25 days after the procedure for the next cycle of chemotherapy. Before admission, he reported a severe coughing episode with cyanosis while eating at home, followed by an episode of suspected foreign body ingestion. Chest radiography and CT performed upon readmission revealed an ingested tracheal stent in the oesophagus (Figure 2A,B). Endoscopic evaluation with both upper gastrointestinal endoscopy and bronchoscopy confirmed the absence of the tracheal stent and identified its presence in the oesophagus (Figure 2C).

The stent was successfully retrieved using the overtube‐assisted technique (Figure 2D). Subsequent endoscopic evaluation revealed two oesophageal ulcers, but no evidence of a tracheoesophageal fistula.

## 3 Discussion

Tracheal stent migration is a well‐documented complication of airway stenting, occurring in approximately 5%–17% of cases, depending on the stent type and underlying pathology [1]. This complication may result from suboptimal stent selection (inappropriate size or length) or patient‐specific factors, including vigorous coughing, dynamic respiratory movements, and tumour regression following oncological treatment. Migration frequently occurs within a few months of stent placement, often before patients develop clinical symptoms, emphasising the necessity for prompt follow‐up assessments to prevent potentially serious complications, such as stent ingestion.

To date, only two cases of tracheal stent ingestion have been documented in the literature, both involving silicone stents [2, 3]. Silicone stents inherently demonstrate a greater migration propensity because of their non‐expandable structure and minimal tissue integration. In the case reported by Vikash et al. [3], a Y‐shaped silicone stent was dislodged and subsequently ingested after an episode of severe coughing. Although silicone stents typically feature external protrusions that are designed to enhance friction with the airway wall, these modifications may be insufficient when airway deformation occurs. The higher migration rates observed with silicone stents than with metallic or hybrid stents primarily reflect their structural characteristics.

To the best of our knowledge, this is the first documented instance of hybrid stent ingestion, raising important questions regarding the limitations of the current antimigration designs. Hybrid stents were developed to combine the structural advantages of metallic stents with the removability of silicone models. Unlike silicone models, hybrid stents exert a continuous outward radial force against the airway wall and often incorporate specific antimigration features, including scale‐like fins along the strut section and flared ends, to increase surface contact. Despite these engineering innovations, our case demonstrates that such design features may not fully counteract dynamic anatomical changes in the airway.

In this case, the migration mechanism warrants further attention. In patients with intraluminal tumours or mixed growth patterns, irregular airway surfaces often provide natural anchorage, particularly across the longer affected segments. Conversely, in cases of pure extraluminal compression, the airway lumen remains relatively smooth, thereby offering minimal friction resistance. In the present case, tumour regression after chemotherapy reduced external compression, increased the airway diameter, and lowered tissue resistance, collectively leading to stent migration and subsequent ingestion.

However, an optimal surveillance protocol for airway stent placement remains unclear. While Hans et al. recommended bronchoscopic evaluation at 4–6 weeks post‐placement for the early detection of stent‐related complications [4], this interval may be inadequate in cases of malignancy‐associated extrinsic compression that can change rapidly. Although bronchoscopy remains the gold standard for airway evaluation, CT serves as a complementary noninvasive tool for detecting early stent complications, including stent migration [5]. Our case demonstrates the value of CT in the diagnosis of stent ingestion. Regardless of the modality employed, early follow‐up should be considered for patients with anticipated rapid tumour regression.

In conclusion, this case highlights the necessity for individualised follow‐up based on patient‐specific factors and pathological dynamics, irrespective of the stent type. Future efforts should focus on establishing standardised surveillance plans for patients with dynamic airway pathologies requiring stenting.

## Author Contributions

Yuya Ishikawa, Hironori Ishibashi, Yukitaka Sato, Yuri Sumi, Ayaka Asakawa, and Ryo Wakejima managed the patient and conceptualised the report. Yuya Ishikawa, Hironori Ishibashi, and Kenichi Okubo conducted the literature review and drafted the manuscript. All authors reviewed and approved the final version of the manuscript.

## Ethics Statement

The authors have nothing to report.

## Consent

The authors declare that written informed consent was obtained for the publication of this manuscript and accompanying images using the consent form provided by the Journal.

## Conflicts of Interest

The authors declare no conflicts of interest.

## Data Availability

Data sharing not applicable to this article as no datasets were generated or analysed during the current study.
